# Exploring feasibility criteria for stereotactic radiosurgical treatment of multiple brain metastases using five linac machines

**DOI:** 10.1002/acm2.14413

**Published:** 2024-06-24

**Authors:** Yusuke Sakai, Kazuki Kubo, Hajime Monzen, Yoshihiro Ueda, Masao Tanooka, Masayoshi Miyazaki, Kentaro Ishii, Ryu Kawamorita

**Affiliations:** ^1^ Department of Medical Physics, Graduate School of Medical Sciences Kindai University Osakasayama Osaka Japan; ^2^ Department of Radiotherapy Takarazuka City Hospital Takarazuka Hyogo Japan; ^3^ Department of Radiation Oncology Osaka International Cancer Institute Chuo‐ku Osaka Japan; ^4^ Department of Radiation Oncology Tane General Hospital Nishi‐ku Osaka Japan

**Keywords:** brain metastasis, SRS, surface area, VMAT

## Abstract

**Purpose:**

This study aimed to find descriptors that correlates with normal brain dose to determine the feasibility of performing fractionated stereotactic radiosurgery (SRS) for multiple brain metastases (BMs) using five linac machines.

**Methods:**

Thirty‐two patients with 1–30 BMs were enrolled. Treatment plans were created using TrueBeam, Novalis Tx, TrueBeam Edge, Halcyon, and Tomotherapy linacs. The sum of all planning target volumes (PTVs) was defined as PTV_all_, and the brain region excluding PTV_all_ was defined as normal brain. The total surface area (TSA) of the PTV was calculated from the sum of the surface areas of the equivalent spheres for each PTV. Volumes receiving more than 5, 12, and 18 Gy (*V*_5Gy_, *V*_12Gy_, and *V*_18Gy_, respectively) were used for evaluation of normal brain dose. Correlations between normal brain dose and each tumor characteristic (number, PTV_all_, and TSA) were investigated using the Spearman rank correlation coefficient.

**Results:**

Correlations between each characteristic and normal brain dose were statistically significant (*p* < 0.05) across all machines. The correlation coefficients between each characteristic and *V*_18Gy_ for the five machines were as follows: tumor number, 0.39–0.60; PTV_all_, 0.79–0.93; TSA, 0.93–0.99. The fit equations between TSA and *V*_18Gy_ exhibited high coefficients of determination, ranging from 0.92 to 0.99 across five machines.

**Conclusion:**

This study devised fractionated SRS plans using for 1–30 BMs across five linac machines to find descriptors for determining SRS feasibility based on normal brain dose. TSA proved to be a promising descriptor of SRS feasibility for treating multiple BMs.

## 1 INTRODUCTION

Brain metastases (BMs) occur in 20%−40% of all cancer patients.^1^ Radiotherapy is considered an effective treatment for BMs, with options including stereotactic radiosurgery (SRS) and whole‐brain radiotherapy. SRS has been chosen for patients with a limited number of BMs (e.g., ≤ 4).^2^, ^3^ SRS provides high local control rates compared with whole‐brain radiotherapy and minimizes side effects such as neurocognitive function decline and radiation‐induced hair loss.^3^, ^4^, ^5^ In recent years, several studies reported the potential to expand the application of SRS to patients with 10 or more BMs.^6^, ^7^, ^8^ Gamma knife has been main approach for SRS, but advancements in linear accelerator technology have also made linear accelerator (linac)‐based SRS feasible.^9^, ^10^ Currently, various linac machines enable SRS and fractionated SRS (fSRS) for BM treatment using volumetric modulated arc therapy (VMAT).^11^, ^12^

The primary concern associated with SRS is radiation‐induced brain necrosis, which strongly correlates with volumes of normal brain receiving specific doses (e.g., volume receiving more than 12 and 18 Gy (*V*_12Gy_ and *V*_18Gy_) for single‐ and multi‐fractionated SRS, respectively).^13^, ^14^, ^15^ Thus, normal brain dose is a crucial factor in judging the feasibility of SRS. Several tumor characteristics are associated with normal brain dose. For example, normal brain dose increases commensurately with the number and total volume of tumors.^16^, ^17^, ^18^, ^19^ [Correction added on July 26, 2024 after first online publication: reference 20 is deleted.] In addition, the intermediate dose (50% of prescribed dose) spill on normal brain depends on the surface area of the planning target volume (PTV).^20^ [Correction added on July 26, 2024 after first online publication: reference 21 is deleted.] As a feasibility criterion for SRS, Takahashi et al. recommended cases with fewer than seven tumors on the basis of dosimetric parameters of normal brain^16^; however, they used non‐VMAT‐SRS with a single treatment machine, and their criterion depends on the irradiation method and prescribed dose. Using VMAT‐SRS, several studies have demonstrated that linac machines achieve favorable target coverage and reduce the organ at risk (OAR)‐dose for multiple BMs.^11^, ^12^, ^21^, ^22^ Nevertheless, these studies investigated either a limited number or volume of tumors; thus, the feasibility criterion for SRS remains unclear and may also vary depending on the type of machine. In the clinic, SRS feasibility therefore remains unclear until a plan is created, and it is difficult to choose the suitable treatment machine according to tumor characteristics such as tumor number, volume, and surface area.

The purpose of this study was to identify descriptors that correlates with *V_18Gy_* in normal brain to determine the feasibility of using SRS for multiple BMs. Utilizing data for patients with 1−30 BMs, we created VMAT‐fSRS plans using five different linac machines and considered factors such as the number, volume, and surface area of tumors.

## 2 MATERIALS AND METHODS

### 2.1 Patient selection and planning

We utilized a dataset comprising 32 patients who underwent radiotherapy for BMs between January 2012 and January 2023 (Table 1). The median number of BMs was four (range: 1−30), and the median total PTV was 11.2 cm^3^ (range: 0.7−56.3 cm^3^). Computed tomography (CT) images were acquired with 1.25 mm slices using an Optima CT580W CT scanner (GE Medical Systems, Waukesha WI, USA) and transferred to a treatment planning system (TPS; Eclipse ver. 15.6, Varian Medical Systems, Palo Alto, CA). The CT images were fused with three‐dimensional contrast‐enhanced T1‐weighted magnetic resonance images to delineate the contour of the gross tumor volume, which was then radially expanded by 1 mm to create the PTV. The contours of the optic nerve, optic chiasm, brainstem, and normal brain were delineated as OARs. PTV_all_ was defined as the sum of all PTVs, and normal brain was defined as the brain region excluding PTV_all_. DICOM data for CT images and structures were sent to each facility. Treatment plans were created using five different linacs: TrueBeam (Varian Medical Systems, Palo Alto, CA), Novalis Tx (Varian Medical Systems, Palo Alto, CA, and BrainLAB, Feldkirchen, Germany), TrueBeam Edge (Varian Medical Systems, Palo Alto, CA), Halcyon (Varian Medical Systems, Palo Alto, CA), and TomoTherapy Radixact X9 (Accuray Incorporated, CA, USA). VMAT delivers intensity‐modulated doses while simultaneously varying multi‐leaf collimator (MLC) positions, dose rate, and gantry rotation speed. HyperArc (equipped on TrueBeam Edge) assists users by automatically selecting an optimal mono‐isocenter, collimator angles, and providing the most conformal plan while minimizing low dose spillage into the surrounding normal brain structures.^22^ TrueBeam and Novalis Tx are c‐arm based linac, with isocentric couch rotation, enabling non‐coplanar irradiation. Halcyon is a VMAT instrument of an O‐ring type linac with a rapid rotation speed of four revolutions per minute. Tomotherapy is also an O‐ring type linac machine, delivering fan‐beam radiation simultaneously with continuous couch movement along the *z*‐axis. Both Halcyon and Tomotherapy are capable of providing only coplanar irradiation. Tomotherapy utilized the helical Tomotherapy delivery mode for all plans, employing a technique that involves continuous gantry rotations around the patient. Each patient's tumors were treated within a single plan. Single‐isocenter VMAT plans were created for all treatment machines except the Tomotherapy system. Table 2 shows the machine information and the plan settings clinically utilized at each facility. For TrueBeam, Novalis‐Tx, and Halcyon systems, the collimator angle was manually set by the planner for each arc to prevent island blocking,^23^ and it was automatically set using the HyperArc software for the TrueBeam Edge system. [Correction added on July 26, 2024 after first online publication: reference 24 is deleted and 23 is included.] TrueBeam and TrueBeam Edge utilized the jaw tracking technique which can reduce the radiation transmission through the MLCs. Three different planners were included in this study, and all plans for one treatment machine were created using the same planner. A radiation dose of 27 Gy, delivered in three fractions, was prescribed such that at least 95% of PTV_all_ (*D*_95%_) received 100% of the prescription dose.^24^ [Correction added on July 26, 2024 after first online publication: reference 25 is deleted and 24 is included.] The maximum dose (*D*_max_) for PTV_all_ was 125%−140%. Dose constraints for each OAR were as follows: *V*_18Gy_ < 30.2 cm^3^ for normal brain; *D*_max_ < 23 Gy and *V*_18Gy_ < 0.5 cm^3^ for brainstem; and *D*_max_ < 17.4 Gy and *V*_15.3 Gy_ < 0.2 cm^3^ for the optic nerve and chiasm. Tumor coverage was prioritized over normal brain and brainstem dose constraints. The tumor volume and number were obtained from the TPS. Since typical metastatic brain tumors are spherical in shape,^25^ we calculated the surface area of tumors under this assumption. [Correction added on July 26, 2024 after first online publication: reference 26 is deleted and 25 is included.] For the Eclipse system, it is possible to display the equivalent sphere diameter for each structure. We calculated the surface area of each PTV using its equivalent diameter and then summed all surface areas to obtain the total surface area (TSA) of PTV_all_.

**TABLE 1:** Data for 32 patients with BMs. PTV_all_ is the sum of the PTVs of all tumors.

Patient	Number of tumors	PTV of each tumor (cm^3^)	PTV_all_ (cm^3^)
1	1	19.9	19.9
2	1	2.9	2.9
3	1	36.1	36.1
4	2	41.9	41.9
5	2	0.9−5.4	6.3
6	2	0.3−0.4	0.7
7	2	0.4−0.6	1.0
8	3	0.3−4.1	5.0
9	3	2.3−39.5	51.4
10	3	0.5−1.1	2.2
11	4	0.3−1.3	3.0
12	4	0.2−9.2	11.0
13	4	0.4−1.3	3.1
14	4	0.2−32.2	33.5
15	4	0.4−23.0	27
16	4	2.5−9.3	24.9
17	4	0.3−24.5	25.8
18	5	0.3−10.6	17.9
19	6	0.2−0.5	1.8
20	6	0.4−1.1	3.6
21	7	0.1−5.5	7.2
22	7	0.2−1.6	4.6
23	8	0.1−5.1	11.4
24	10	0.2−9.5	21.8
25	10	0.3−20.5	38.8
26	11	0.2−8.3	11.9
27	11	0.2−4.3	8.4
28	15	0.2−7.5	14.9
29	15	0.1−0.4	3.1
30	17	0.3−1.7	13.5
31	20	0.2−0.8	9.0
32	30	0.2−11.9	56.3

*Note*: The data were obtained from the TPS.

Abbreviations: BMs, brain metastases; PTV, planning target volume; TPS, treatment planning system.

**TABLE 2:** Machines and parameters used to create plans in this study.

	TrueBeam	Novalis Tx	Edge	Halcyon	Tomotherapy
Irradiation method	VMAT	VMAT	HyperArc VMAT	VMAT	Helical Tomotherapy
X‐ray energy	10 MV‐FFF	6 MV	6 MV‐FFF	6 MV‐FFF	6 MV‐FFF
MLC thickness (mm)	5	2.5	2.5	10	6.25
TPS	Eclipse	Eclipse	Eclipse	Eclipse	Precision
Calculation algorithm	AXB	AXB	AAA	AXB	CCC
Dose calculation grid (mm)	1	1	1.25	2	1
Determination method of collimator angle	Manual	Manual	Auto	Manual	–
Plan settings	Coplanar 1 full arc (0 degrees) Non‐coplanar 3 half arcs (45, 135, 90, or 270 degrees)			Coplanar 4 or 5 arcs	Jaw width: 1 cm Pitch: 0.08−0.2

*Note*: These plan settings are used in clinical practice at each facility.

Abbreviations: AAA, anisotropic analytical algorithm; AXB, Acuros XB; CCC, collapsed cone convolution; FFF, flattening filter‐free; TPS, treatment planning system; VMAT volumetric modulated arc therapy.

### 2.2 Dosimetric plan evaluation

The following dosimetric parameters were evaluated: mean dose (*D*_mean_), *D*_2%_, and *D*_98%_; Paddick conformity index (CI) and gradient index (GI) for PTV_all_; and *V*_5Gy_, *V*_12Gy_, and *V*_18Gy_ for normal brain. The CI was calculated as follows:

$$\mathrm{CI}={\left(V_{\mathrm{PI},\mathrm{PTV}}\right)}^{2}/\left(V_{\mathrm{PTV}}\times V_{\mathrm{PI}}\right)$$

where *V*_PI, PTV_ is the PTV covered by 100% of the prescription dose, *V*_PTV_ is the PTV, and *V*_PI_ is the volume covered by 100% of the prescription dose. The GI was calculated using

$$\mathrm{GI}={\mathrm{PV}}_{50\%}/{\mathrm{PV}}_{100\%}$$

where PV_50%_ is the volume covered by 50% of the prescription dose and PV_100%_ is the volume covered by 100% of prescription dose.

All plans were imported into the same TPS (Eclipse ver. 15.6, Varian Medical Systems, Palo Alto, CA), and the dosimetric parameters were analyzed. The correlations between the normal brain dose and various factors including the number of tumors, PTV_all_, and TSA were investigated. Using SPSS Statistics v28.0 (IBM Inc., Armonk, USA), we calculated the Spearman rank correlation coefficient (*ρ*). A *p*‐value less than 0.05 was considered to denote statistical significance. Either a linear or quadratic polynomial approximation was used, depending on which best fit the data. The coefficient of determination (*R* ^2^) was used as an indicator of goodness of fit of the model.

## 3 RESULTS

Table 3 shows the dosimetric results for PTV_all_ and normal brain. All dose constraints for OARs were satisfied except for brainstem containing tumors and *V*_18Gy_ of the normal brain.

**TABLE 3:** Dosimetric parameters for PTV_all_ and normal brain for five machine plans.

	TrueBeam	Novalis Tx	Edge	Halcyon	Tomotherapy
PTV_all_	Median [range]				
*D*_mean_ (%)	113.6 [109.0−118.3]	112.8 [109.4−115.9]	118.0 [114.7−125.4]	114.8 [108.5−117.4]	115.3 [110.3−119.6]
*D*_2%_ (%)	130.9 [121.0−136.2]	126.8 [119.9−134.9]	135.2 [132.0−140.5]	132.2 [120.7−138.4]	133.6 [125.3−138.8]
*D*_98%_ (%)	98.0 [97.4−98.7]	97.9 [92.2−98.6]	97.7 [96.1−98.8]	97.7 [96.5−98.2]	97.5 [96.3−98.6]
Paddick CI	0.91 [0.80−0.95]	0.92 [0.83−0.96]	0.94 [0.81−1.00]	0.84 [0.59−0.95]	0.87 [0.65−0.95]
GI	5.1 [2.3−17.3]	4.6 [2.3−10.2]	3.5 [2.2−6.6]	6.6 [3.1−17.1]	6.2 [3.2−12.5]
Normal brain					
*V*_5Gy_ (cm^3^)	241.7 [19.7−1203.4]	234.8 [10.8−1142.8]	232.7 [11.1−1237.5]	275.5 [39.3−958.6]	349.5 [38.8−1206.2]
*V*_12Gy_ (cm^3^)	53.5 [4.3−435.2]	47.1 [3.2−509.7]	33.1 [2.6−315.4]	65.3 [8.7−428.3]	99.2 [10.6−800.4]
*V*_18Gy_ (cm^3^)	19.8 [1.7−180.2]	16.7 [1.3−162.8]	11.5 [1.0−105.8]	25.0 [3.3−163.9]	23.6 [2.7−137.9]
*D*_mean_ (%)	14.5 [2.4−39.1]	14.4 [2.3−37.3]	13.2 [1.8−35.5]	12.4 [2.2−35.5]	15.2 [2.6−42.9]
maximum permissible TSA (cm^2^)	65.1	70.3	87.2	50.6	56.4

Abbreviations: GI, gradient index; Paddick CI, Paddick conformity index; PTV; planning target volumes; TSA, Total surface area.

**FIGURE 1:**
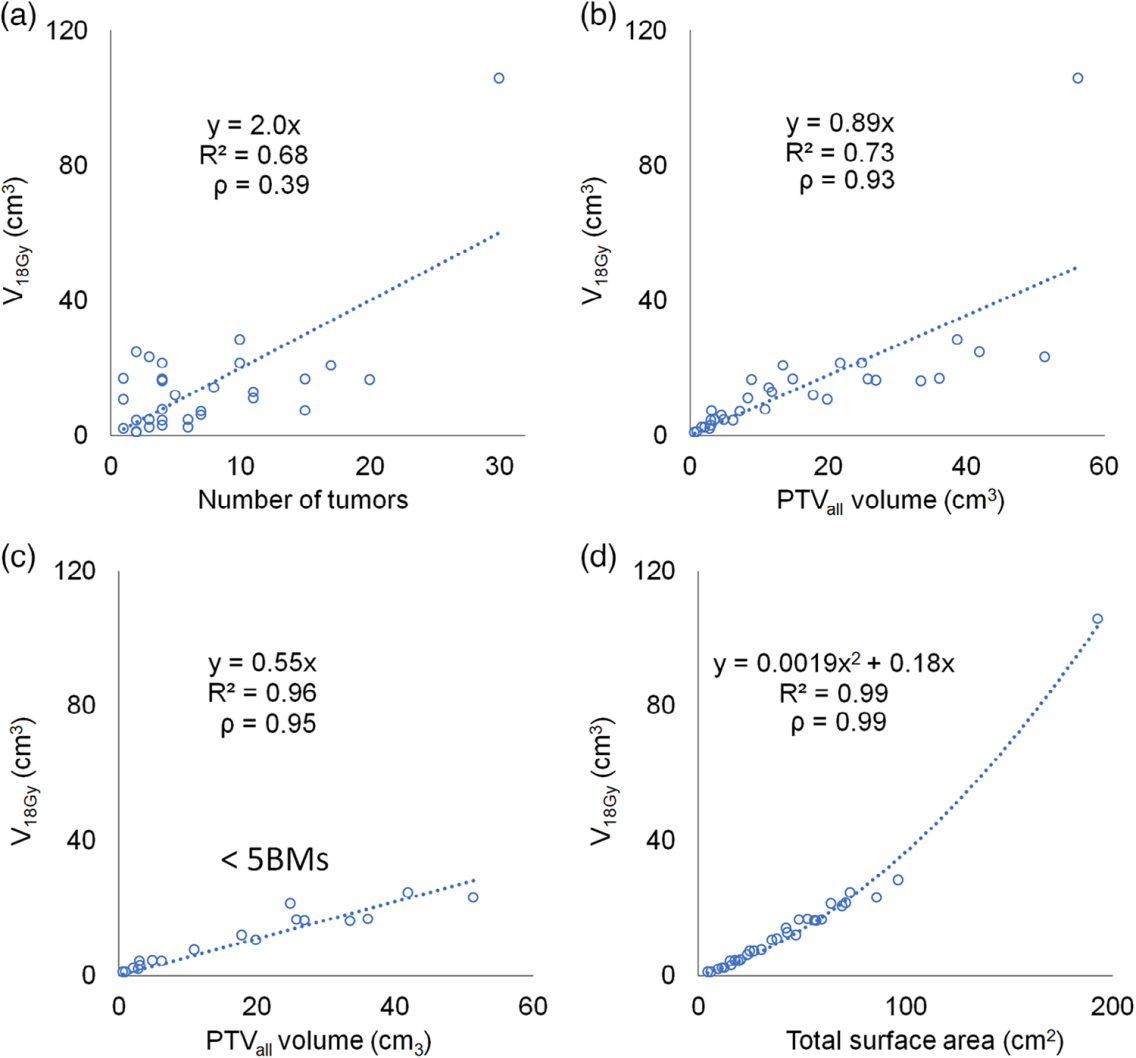
Volume of normal brain receiving more than 18 Gy (*V*_18Gy_) from the TrueBeam edge system as a function of (a) number of tumors, (b) PTV_all_ for all patients, (c) PTV_all_ only for patients with five or fewer tumors, and (d) TSA. The dashed curves represent the approximate straight‐line fits. PTV, planning target volume; TSA, total surface area.

Figure 1 shows the dependences of tumor number, PTV_all_, and TSA on *V*_18Gy_ of normal brain for the Edge system. TSA had the highest correlation with *V_18Gy_* compared with the other characteristics, and this trend was the same for five machines. The coefficients of correlation between each characteristic and *V*_18Gy_ for the five machines were as follows: tumor number, 0.39−0.60; PTV_all_, 0.79−0.93; TSA, 0.93−0.99. When limited to patients with five or fewer tumors, coefficients of correlation between *V*_18Gy_ and PTV_all_ were in the 0.93−0.96 range. The correlations between *V*_18Gy_ and tumor number, PTV_all_, and TSA were statistically significant (*p* < 0.05) across all machines. Among the normal brain dose indices, correlation with TSA was highest for *V*_18Gy_ and lowest for *V*_5Gy_ across all machines.

**FIGURE 2:**
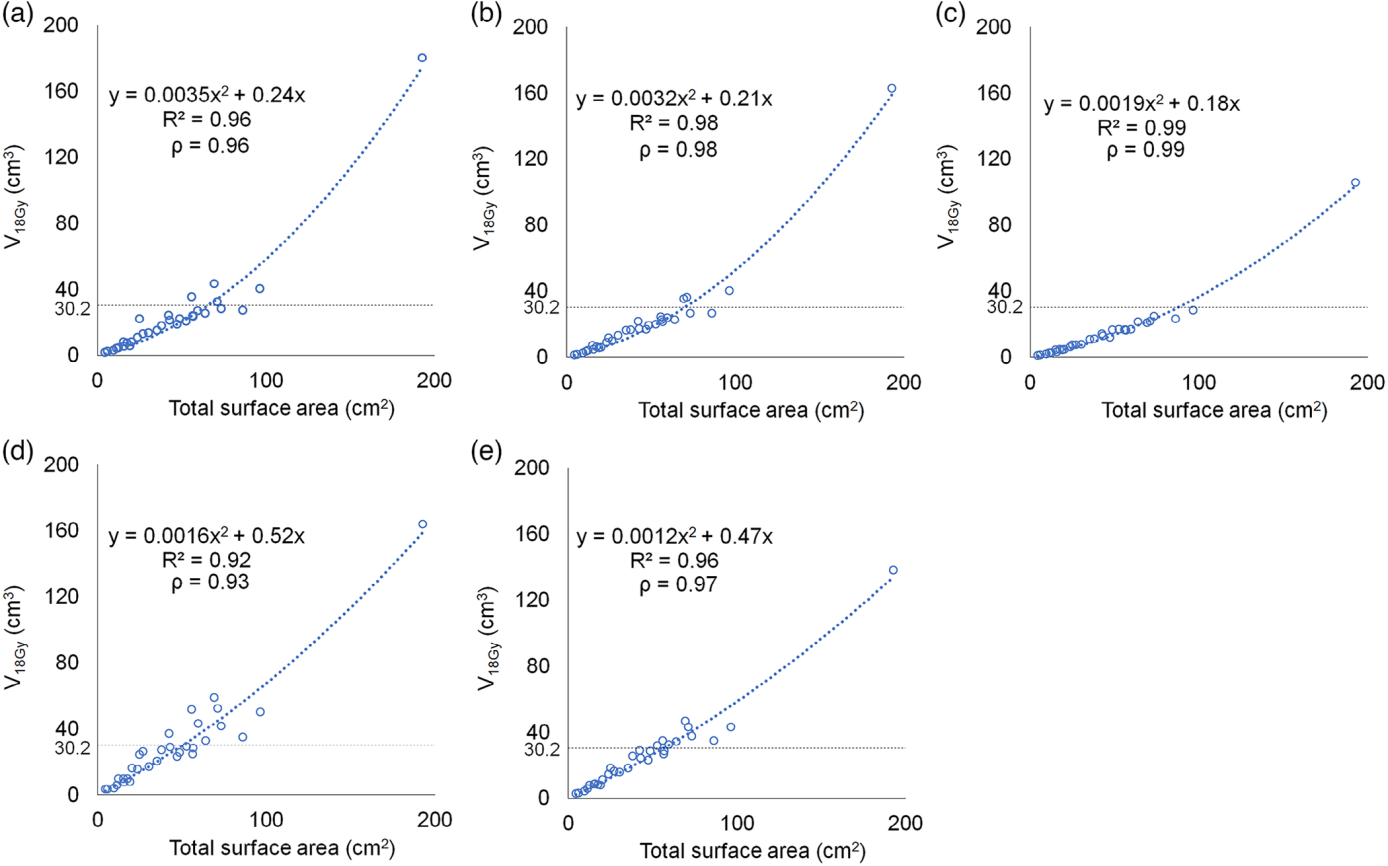
Dependence of TSA on *V*_18Gy_ for normal brain using (a) TrueBeam, (b) Novalis Tx, (c) TrueBeam Edge, (d) Halcyon, and (e) Tomotherapy systems. The dashed curves represent the approximate straight‐line fits. The dashed horizontal lines indicate the 30.2 Gy dose constraint for normal brain.

Figure 2 illustrates the dependence of TSA on *V*_18Gy_ of normal brain for each machine. The coefficients of determination of the corresponding fit equations for the five machines were in the 0.92−0.99 range.

The maximum permissible TSA that satisfies the normal‐brain constraint (*V*_18Gy_ = 30.2 cm^3^) was calculated from the fit equations in Figure 2 as follows: TrueBeam, 65.1 cm^2^; Novalis Tx, 70.3 cm^2^, Edge, 87.2 cm^2^, Halcyon, 50.6 cm^2^, Tomotherapy, 56.4 cm^2^.

## 4 DISCUSSION

In this study, we investigated the relationship between normal brain dose and descriptors such as tumor number, PTV_all_, and TSA to clarify the eligibility criteria for SRS. We investigated five treatment machines to find useful descriptors that are independent of machine type. Significant correlations with normal brain dose indices *V*_5Gy_, *V*_12Gy_, and *V*_18Gy_ were identified for all tumor characteristics (*p* < 0.05). Among them, TSA exhibited the highest correlation with normal brain doses for all five treatment machines. This is thought to be because plans are designed to deliver the prescribed dose to the surface of the PTV, and the 18 Gy isodose line in SRS plans typically follows a rather tight “spherical shell” distribution. On the Eclipse system, it is possible to display the equivalent sphere diameter of a structure, hence TSA can be easily calculated in the clinic. In addition, compared with other parameters, TSA had the smallest range of correlation coefficients across all machines (*ρ* = 0.93−0.99), indicating that it is a useful descriptor regardless of machine type (e.g., coplanar or non‐coplanar). Among normal brain dose indices, *V*_18Gy_ exhibited the highest correlation with TSA, while the low‐dose *V*_5Gy_ parameter had the lowest correlation because of interference from the dose distribution of other tumors and the variable extent of optimization for low‐dose spread.

PTV_all_ is a significant predictor of normal brain dose,^19^, ^26^ and Agostinelli et al. reported an almost perfect linear correlation (*R* ^2^ = 0.97) between *V*_12Gy_ and the PTV.^26^ [Correction added on July 26, 2024 after first online publication: reference 20 is deleted and 19 is included.] However, they investigated cases with only a limited number of tumors. Similarly, in this study, we found *V*_18Gy_ had a strong correlation (*ρ *= 0.93−0.96) with PTV_all_ for cases with five or fewer BMs (Figure 1c); however, including all cases (Figure 1b) resulted in a lower correlation (*ρ* = 0.79−0.93). Therefore, PTV_all_ may not be a suitable predictor of the dose delivered to normal brain in cases with numerous tumors because the correlation depends on the number of such tumors. In contrast, the TSA strongly correlated with *V*_18Gy_ regardless of the number of tumors and value of PTV_all_.

This study focused on the normal brain dose, which is closely related to radiation‐induced brain necrosis.^13^, ^14^, ^15^ The results revealed a strong correlation between the normal brain dose and TSA, and this trend was consistent across five treatment machines. Similarly, Desai et al. showed that the intermediate dose in the normal brain strongly correlates with the surface area of the PTV, which is a characteristic of a treatment modality capable of high conformity regardless of the method of radiation delivery.^20^ Hence, TSA may serve as a useful descriptor of normal brain dose in high‐conformity VMAT‐SRS treatment. In Figure 2, the coefficient of determination was high for all machines (*R* ^2^ = 0.92−0.99), indicating a good fit of the model and thus a consistent relationship between TSA and *V*_18Gy_. By comparing the TSA with past plans in a given facility, it might be possible to predict the normal brain dose, which can be helpful in assessing the feasibility of SRS for cases with various tumor numbers and volumes before creating a treatment plan.

HyperArc allowed the highest maximum permissible TSA among five machines, indicating the greatest sparing of the normal brain. On the other hand, Halcyon and Tomotherapy utilizing only coplanar irradiation resulted in lower sparing. Several studies reported the effectiveness of using noncoplanar beam for intracranial tumor.^27^, ^28^, ^29^ Torizuka et al. showed that VMAT with non‐coplanar irradiation significantly reduced *V*_12Gy_ in normal brain when compared with VMAT with only coplanar irradiation.^29^ One of the purposes of using non‐coplanar beams is to improve dose concentration to the target by reducing the overlap of each beam's irradiation field. However, when the target size is large, it becomes challenging to reduce the overlap of irradiation fields occurring outside the target even when using non‐coplanar beams, diminishing the advantages of employing non‐coplanar beams. Misa et al. reported HyperArc and Halcyon provided the similar plan quality for larger‐sized targets (mean PTV: 34.7 cc/target).^30^ Meanwhile, Agostinelli et al. showed that Tomotherapy resulted in inferior normal brain dose compared with non‐coplanar SRS techniques for small‐sized targets (mean PTV: 5.9 cc/target). In this study, the mean PTV was 2.3cc/target.

A limitation of this study was the limited analysis of cases with large numbers and surface areas of tumors. We included cases with up to 30 tumors, which deviates significantly from the current application of SRS to BMs. In particular, the case with the largest *V*_18Gy_ (105.8 cm^3^) and 30 tumors may influence the model fitting (Figure 1a,b,d). However, regardless of the inclusion of this case, the correlation and determination coefficients between TSA and *V*_18Gy_ were the highest. Additionally, the extremely high *V*_18Gy_ in Figure 1a,b, cannot be explained on the basis of the number or PTV_all_ of tumors. In contrast, in Figure 1d, it is evident that the reason for the case with the high *V*_18Gy_ is its correspondingly high TSA (more than twice that of other cases). TSA has the potential to be applied even to cases with an extremely large number of tumors. To clarify these aspects, it is necessary to analyze additional cases. In addition, since we aimed to conduct clinically relevant assessments, we utilized cumulative *V_xxGy_* and *V_xxGy_* per target was not evaluated. A further limitation of this study was that the relationship between *V*_18Gy_ and TSA will depend on the treatment machine, dose constraints, treatment plan goals and thus on the facility. In this study, the normal brain dose was prioritized when created plans; therefore, it is necessary to establish a *V*_18Gy_ constraint for each facility, as shown in Figure 2. However, once created, this constraint may serve as a criterion for reconsidering plans in cases where *V*_18Gy_ exceeds it.

## 5 CONCLUSION

We created VMAT‐fSRS plans for cases with 1−30 BMs using five linac machines, aiming to find descriptors for determining SRS feasibility based on normal brain dose. Dosimetric plans met OAR constraints except for brainstem containing tumors and *V*_18Gy_ of the normal brain, and a significant correlation existed between TSA and *V*_18Gy_. TSA therefore emerges as a promising indicator of SRS feasibility for treating multiple BMs.

## AUTHOR CONTRIBUTIONS

*Conception and design*: Yusuke Sakai and Kazuki Kubo. *Generation of treatment plan*: Yusuke Sakai, Kazuki Kubo, and Yoshihiro Ueda. Writing, review and/or revision of the manuscript: Yusuke Sakai, Kazuki Kubo, Hajime Monzen, Yoshihiro Ueda, Masao Tanooka, Masayoshi Miyazaki, Kentaro Ishii, and Ryu Kawamorita. All authors read and approved the final manuscript.

## FUNDING INFORMATION

This work was supported partly by Japan Society for the Promotion of Science (JSPS) KAKENHI (Grant No. 23K07194).

## CONFLICT OF INTEREST STATEMENT

The authors declare that they have no conflict of interest.

## ETHICS STATEMENT

This study was approved by the Clinical Research Ethics Review Committee of Tane General Hospital (registration number:2023−21) and followed an opt‐out disclosure process.

## Data Availability

The data that support the findings of this study are available on request from the corresponding author. The data is not publicly available due to privacy or ethical restrictions.

## References

[1] Soffietti R , Rudā R , Mutani R . Management of brain metastases. J Neurol. 2002;249(10):1357‐1369. doi:10.1007/s00415-002-0870-6 12382150

[2] Kocher M , Wittig A , Piroth MD , et al. Stereotactic radiosurgery for treatment of brain metastases. A report of the DEGRO working group on stereotactic radiotherapy. Strahlenther Onkol. 2014;190(6):521‐532. doi:10.1007/s00066-014-0648-7 24715242

[3] Perlow HK , Dibs K , Liu K , et al. Whole‐Brain radiation therapy versus stereotactic radiosurgery for cerebral metastases. Neurosurg Clin N Am. 2020;31(4):565‐573. doi:10.1016/j.nec.2020.06.006 32921352

[4] Brown PD , Jaeckle K , Ballman KV , et al. Effect of radiosurgery alone vs radiosurgery with whole brain radiation therapy on cognitive function in patients with 1 to 3 brain metastases: a randomized clinical trial. JAMA. 2016;316(4):401‐409. doi:10.1001/jama.2016.9839 27458945 PMC5313044

[5] Chang EL , Wefel JS , Hess KR , et al. Neurocognition in patients with brain metastases treated with radiosurgery or radiosurgery plus whole‐brain irradiation: a randomised controlled trial. Lancet Oncol. 2009;10:1037‐1044. 19801201 10.1016/S1470-2045(09)70263-3

[6] Yamamoto M , Serizawa T , Shuto T , et al. Stereotactic radiosurgery for patients with multiple brain metastases (JLGK0901): a multi‐institutional prospective observational study. Lancet Oncol. 2014;15(4):387‐395. doi:10.1016/S1470-2045(14)70061-0 24621620

[7] Yamamoto M , Higuchi Y , Sato Y , Aiyama H , Kasuya H , Barfod BE . Stereotactic radiosurgery for patients with 10 or more brain metastases. Prog Neurol Surg. 2019;34:110‐124. doi:10.1159/000493056 31096244

[8] Rogers SJ , Lomax N , Alonso S , Lazeroms T , Riesterer O . Radiosurgery for five to fifteen brain metastases: a single centre experience and a review of the literature. Front Oncol. 2022;12:866542. doi:10.3389/fonc.2022.866542 35619914 PMC9128547

[9] Albers EAC , de Ruiter MB , van de Poll‐Franse LV , Merckel LG ; Compter A ; Schagen SB . Neurocognitive functioning after gamma knife and LINAC stereotactic radiosurgery in patients with brain metastases. J Neurooncol. 2022;160(3):649‐658. doi:10.1007/s11060-022-04185-3 36454373 PMC9713121

[10] Calugaru E , Whiting Z , Delacruz B , et al. Direct dosimetric comparison of linear accelerator vs. Gamma knife fractionated stereotactic radiotherapy (fSRT) of large brain tumors. Med Dosim. 2023;48(1):31‐36. doi:10.1016/j.meddos.2022.09.006 36503990

[11] Hofmaier J , Bodensohn R , Garny S , et al. Single isocenter stereotactic radiosurgery for patients with multiple brain metastases: dosimetric comparison of VMAT and a dedicated DCAT planning tool. Radiat Oncol. 2019;14(1):103. doi:10.1186/s13014-019-1315-z 31186023 PMC6560766

[12] Liu H , Thomas EM , Li J , et al. Interinstitutional plan quality assessment of 2 linac‐based, single‐isocenter, multiple metastasis radiosurgery techniques. Adv Radiat Oncol. 2020;5(5):1051‐1060. doi:10.1016/j.adro.2019.10.007 33089021 PMC7560574

[13] Minniti G , Scaringi C , Paolini S , et al. Single‐Fraction versus multifraction (3 × 9 Gy) stereotactic radiosurgery for large (>2 cm) brain metastases: a comparative analysis of local control and risk of radiation‐induced brain necrosis. Int J Radiat Oncol Biol Phys. 2016;95(4):1142‐1148. doi:10.1016/j.ijrobp.2016.03.013 27209508

[14] Blonigen BJ , Steinmetz RD , Levin L , Lamba MA , Warnick RE , Breneman JC . Irradiated volume as a predictor of brain radionecrosis after linear accelerator stereotactic radiosurgery. Int J Radiat Oncol Biol Phys. 2010;77(4):996‐1001. doi:10.1016/j.ijrobp.2009.06.006 19783374

[15] Korytko T , Radivoyevitch T , Colussi V , et al. 12 Gy gamma knife radiosurgical volume is a predictor for radiation necrosis in non‐AVM intracranial tumors. Int J Radiat Oncol Biol Phys. 2006;64(2):419‐424. doi:10.1016/j.ijrobp.2005.07.980 16226848

[16] Takahashi M , Narabayashi I , Kuroiwa T , et al. Stereotactic radiosurgery (SRS) for multiple metastatic brain tumors: effects of the number of target tumors on exposure dose in normal brain tissues. Int J Clin Oncol. 2003;8(5):289‐296. doi:10.1007/s10147-003-0331-y 14586753

[17] Raza GH , Capone L , Tini P , et al. Single‐isocenter multiple‐target stereotactic radiosurgery for multiple brain metastases: dosimetric evaluation of two automated treatment planning systems. Radiat Oncol. 2022;17(1):116. doi:10.1186/s13014-022-02086-3 35778741 PMC9250172

[18] Narayanasamy G , Smith A , Van Meter E , Mc Garry R , Molloy JA . Total target volume is a better predictor of whole brain dose from gamma stereotactic radiosurgery than the number, shape, or location of the lesions. Med Phys. 2013;40:091714. doi:10.1118/1.4818825 24007147 PMC4108722

[19] Zhang I , Antone J , Li J , et al. Hippocampal‐sparing and target volume coverage in treating 3 to 10 brain metastases: a comparison of gamma knife, single‐isocenter VMAT, CyberKnife, and Tomotherapy stereotactic radiosurgery. Pract Radiat Oncol. 2017;7(3):183‐189. doi:10.1016/j.prro.2017.01.012 28477798

[20] Desai DD , Johnson EL , Cordrey IL . The surface area effect: how the intermediate dose spill depends on the PTV surface area in SRS. J Appl Clin Med Phys. 2021;22(3):186‐195. doi:10.1002/acm2.13203 PMC7984485 33596329

[21] Ruggieri R , Naccarato S , Mazzola R , et al. Linac‐based radiosurgery for multiple brain metastases: comparison between two mono‐isocenter techniques with multiple non‐coplanar arcs. Radiother Oncol. 2019;132:70‐78. doi:10.1016/j.radonc.2018.11.014 30825972

[22] Ueda Y , Ohira S , Yamazaki H , et al. Dosimetric performance of two linear accelerator‐based radiosurgery systems to treat single and multiplebrain metastases. Br J Radiol. 2019;92(1100):20190004. doi:10.1259/bjr.20190004 31188018 PMC6724620

[23] Wu Q , Snyder KC , Liu C , et al. Optimization of treatment geometry to reduce normal brain dose in radiosurgery of multiple brain metastases with single‐isocenter volumetric modulated arc therapy. Sci Rep. 2016;6:34511. doi:10.1038/srep34511 27688047 PMC5043272

[24] Meeks SL , Mercado CE , Popple RA , et al. Practical considerations for single isocenter LINAC radiosurgery of multiple brain metastases. Pract Radiat Oncol. 2022;12(3):195‐199. doi:10.1016/j.prro.2021.09.007 34619373

[25] Warnick RE , Darakchiev BJ , Breneman JC . Stereotactic radiosurgery for patients with solid brain metastases: current status. J Neurooncol. 2004;69(1‐3):125‐137. 15527085 10.1023/b:neon.0000041876.90641.96

[26] Agostinelli S , Garelli S , Gusinu M , et al. Dosimetric analysis of Tomotherapy‐based intracranial stereotactic radiosurgery of brain metastasis. Phys Med. 2018;52:48‐55. doi:10.1016/j.ejmp.2018.06.632 30139609

[27] A Uto M , Mizowaki T , Ogura K , Hiraoka M . Non‐coplanar volumetric‐modulated arc therapy (VMAT) for craniopharyngiomas reduces radiation doses to the bilateral hippocampus: a planning study comparing dynamic conformal arc therapy, coplanar VMAT, and non‐coplanar VMAT. Radiat Oncol. 2016;11:86. doi:10.1186/s13014-016-0659-x 27338798 PMC4918038

[28] Panet‐Raymond V , Ansbacher W , Zavgorodni S , et al. Coplanar versus noncoplanar intensity‐modulated radiation therapy (IMRT) and volumetric‐modulated arc therapy (VMAT) treatment planning for fronto‐temporal high‐grade glioma. J Appl Clin Med Phys. 2012;13(4):3826. doi:10.1120/jacmp.v13i4.3826 22766954 PMC5716518

[29] Torizuka D , Uto M , Takehana K , Mizowaki T . Dosimetric comparison among dynamic conformal arc therapy, coplanar and non‐coplanar volumetric modulated arc therapy for single brain metastasis. J Radiat Res. 2021;62(6):1114‐1119.rrab092. 10.1093/jrr/rrab092 34604907

[30] Misa J , McCarthy S , Clair WS , Pokhrel D . Stereotactic radiotherapy of intracranial tumor beds on a ring‐mounted Halcyon LINAC. J Appl Clin Med Phys. 2024:e14281. doi:10.1002/acm2.14281 38277473 PMC11163492

